# Molecular imaging of lymphatic organs provides prognostic value after acute myocardial infarction

**DOI:** 10.1007/s00259-026-07809-2

**Published:** 2026-03-11

**Authors:** Theresa Reiter, Anna-Lena Dörrler, Natalie Hasenauer, Sebastian E. Serfling, Takahiro Higuchi, Nils Kraus, Wolfgang R. Bauer, Willibald Hochholzer, Gustavo Ramos, Samuel Samnick, Ulrich Hofmann, Stefan Frantz, Andreas K. Buck, Aleksander Kosmala, Rudolf A. Werner

**Affiliations:** 1 Department of Electrophysiology, German Heart Center Munich, Technical University of Munich, Munich, Germany; 2 Department of Nuclear Medicine, University Hospital Würzburg, Würzburg, Germany; 3 Faculty of Medicine, Dentistry and Pharmaceutical Sciences, Okayama University, Okayama, Japan; 4 Comprehensive Heart Failure Center, University Hospital Würzburg, Würzburg, Germany; 5 Department of Internal Medicine I, University Hospital Würzburg, Würzburg, Germany; 6 Department of Cardiology and Intensive Care Medicine, Klinikum Würzburg Mitte, Würzburg, Germany; 7 Department of Nuclear Medicine, LMU University Hospital, LMU Munich, Marchioninistr. 15, 81377 Munich, Munich Germany

**Keywords:** PET, CXCR4, Spleen, Lymphatic organs, AMI, Myocardial infarction

## Abstract

**Introduction:**

Acute myocardial infarction (AMI) triggers local inflammation in the injured myocardium, followed by a systemic inflammatory response of lymphatic organs. This prospective trial (NCT05519735) focused on molecular imaging of lymphatic organs (spleen, bone marrow, and heart-draining lymph nodes) and aimed to determine whether uptake in such remote organs identifies individuals predisposed to functional recovery during follow-up after AMI.

**Methods:**

41 timely re-perfused ST-elevation AMI patients received baseline C-X-C motif chemokine receptor 4 directed ^68^Ga-PentixaFor PET, a radiotracer targeting a broad spectrum of leukocytes. To determine left ventricular ejection fraction (LVEF) and infarct size, cardiac magnetic resonance imaging was conducted at baseline and repeated after six (follow-up (FU) 1, available in 38/41) and twelve months (FU 2, available in 36/41). As endpoint, an LVEF increase of ≥ 5% relative to baseline was defined as short- (at FU 1) and long-term functional (at FU 2) recovery. We also determined association of ^68^Ga-PentixaFor uptake in lymphatic organs with outcome relative to established clinical and imaging biomarkers.

**Results:**

LVEF at baseline was 50.4 ± 8.7% (range 34–65%). At FU 1, LVEF improved significantly (54.2 ± 7.1%, *P* = 0.0004 vs. baseline) and the endpoint was recorded in 21/38 patients. Univariate analysis identified baseline LVEF (Odds Ratio (OR), 0.80, *P* = 0.002) and uptake derived from heart-draining lymph nodes (OR, 0.21, *P* = 0.03) as predictor of functional recovery, while only LVEF reached significance at multivariate analysis (OR, 0.72, *P* = 0.007). At FU 2, LVEF also improved to 54.3 ± 7.3% relative to baseline (*P* = 0.006) and the endpoint was met in 21/36 patients. Baseline LVEF (OR 0.84, *P* = 0.005) and splenic PET signal (OR, 2.46, *P* = 0.04) provided prognostic value at univariate analysis. Both parameters remained significant at multivariate outcome analysis (LVEF: OR, 0.73, *P* = 0.01; spleen: OR, 4.17, *P* = 0.04), indicating that baseline LVEF and splenic uptake are prognostic for improved long-term functional outcome. Established risk factors of cardiac damage (infarct size) or inflammation (C-reactive protein, white blood cell counts) failed to reach significance for functional recovery at both follow-up time-points.

**Conclusions:**

Inflammatory imaging in lymphatic organs may provide a complementary in-vivo biomarker for functional recovery and seems to be more strongly associated with outcome relative to standard markers of cardiac damage or inflammation.

**Supplementary Information:**

The online version contains supplementary material available at 10.1007/s00259-026-07809-2.

## Introduction

Recent years have witnessed an increasing amount of evidence for immune modulating therapeutic interventions after myocardial infarction (MI) [1–3]. However, there is an unmet need in biomarker or imaging-guided approaches for clinical decision-making on immune-modulatory therapy or identifying high-risk individuals prone to cardiac functional changes during follow-up (FU). Cardiac magnetic resonance imaging (CMR) is the method of choice to determine the current cardiac functional status and multiple trials have reported on respective associations between CMR-derived parameters and outcome [4, 5]. Despite those clinical benefits, CMR has limited specificity for detecting inflammation in the heart, while MR-imaging of immune-activity in secondary lymphatic organs has not been established. The PET tracer principle allows for evaluating the underlying pathophysiology at the target site (infarcted myocardium) and remote organs, which are involved in the systemic immune response [6].

Among others, C-X-C motif chemokine receptor 4 (CXCR4) is crucially involved in the tight orchestration of recruiting and homing of stem cells to the under-perfused cardiomyocytes after acute MI (AMI) [7]. In the injured heart, CXCR4 is not confined to one cell type, but lymphocytes account for the vast majority of CXCR4-positive cells in lymph nodes (LN, > 90%) [8]. The broad expression of CXCR4 in bone marrow-associated progenitor cells or natural killer cells also provides a rationale for monitoring chemokine receptor expression in the spleen and bone marrow after an acute event [9, 10]. Due to its considerable low cardiac background activity, ^68^Ga-PentixaFor, a PET radiotracer targeting CXCR4 on a broad spectrum of leukocytes, has been investigated in recent years in varying pre- and clinical scenarios post-MI [11]. For instance, a recent retrospective investigation post-AMI showed that the cardiac infarct uptake area serves as predictor for left ventricular ejection fraction (LVEF) change during a single FU time-point [12]. As radiotracers, however, are applied systemically, chemokine receptor expression of organs of interest including heart-draining LN, spleen and bone marrow can also be determined through a whole-body PET/CT approach [11].

As such, we hypothesized that imaging of CXCR4 expression in organs involved in the systemic immune response can provide incremental prognostic performance relative to other established clinical parameters for functional recovery in first AMI patients. While retrospective investigations focused on the infarct territory [12, 13], the present prospective trial aimed to determine whether CXCR4-targeted PET of extra-cardiac organs involved in the immune response identifies high-risk individuals prone to myocardial functional changes during standardized short- and long-term FU six and twelve months after AMI.

## Materials and methods

*Study Design and Participants.* This prospective trial (NCT05519735) enrolled 41 patients (12 females, 59 ± 9 years) which received CXCR4-directed ^68^Ga-PentixaFor within 8 days after the acute ST-elevation, single-vessel MI, allocated to an early (2–4 days) or late imaging group (5–8 d) based on tracer availability. All patients had received invasive coronary angiography and revascularization of the culprit lesion within 120 min after diagnosis. Further inclusion criteria included minimum age of 18 years and immediate catheterization, along with stable clinical course. Exclusion criteria can also be found in [14]. Patients also received CMR at baseline after AMI. To determine early and long-term functional recovery, CMR was then repeated at six (FU 1) and twelve months (FU 2), thereby allowing for standardized re-assessments with fixed time-points. We recorded standard laboratory values of cardiac damage, including peak Troponin T (in ng/ml), peak creatine kinase (in U/l), peak lactate dehydrogenase (in U/l), and N-terminal prohormone of brain natriuretic peptide (in pg/ml). At day of PET imaging, creatinine (in mg/dl) and systemic inflammatory markers, including white blood cells (in x1000/µl) and C-reactive protein (CRP, in mg/dl), were also collected from the blood. Patients gave written informed consent prior to the study and the local ethical committee approved this trial (#192/21). Details on patient characteristics can be found in Supplementary Table 1.

*Imaging and Outcome.* ^68^Ga-PentixaFor was produced in-house [15], following good manufacturing practice by using a Scintomics synthesis module (Gräfelfing, Germany) and single-use cassette kits provided by ABX (Radeberg, Germany). Image acquisition took place 45 min after injection of 145 ± 13 MBq ^68^Ga-PentixaFor, with a field-of-view ranging from the cervical region to the spleen. In 3D mode, measurements were taken for 2 min per bed position, and image reconstruction was performed using an ordered subset expectation maximization algorithm (attenuation-weighted ordered subsets expectation maximization algorithm; 4 iterations, 8 subsets). Static PET images over 20 min were conducted on either a Biograph mCT 64 or 128 PET/CT (Siemens, Erlangen, Germany). Guided by PET/CT, we set volumes of interest on the PET portion in the infarcted and remote myocardium, spleen, bone marrow of the vertebral bodies and heart-draining and remote LN in the axillary region (with no discernible tracer uptake) to determine peak standardized uptake values (SUV) [11]. As described previously [13], we then calculated the uptake ratio of infarcted/remote myocardium (referred to as infarct) and heart-draining/remote LN. For the latter, we then determined the median ratios of all investigated LNs (referred to as LN). Low-dose CT was also conducted for attenuation correction and for anatomical guidance of LN stations (left or right hilar, infra-carinal, left or right mediastinal) and to determine number of investigated LN.

All CMR scans were performed on a 3.0T clinical MRI scanner (Achieva DS, Philips Healthcare, Best, The Netherlands) and performed following current recommendations [16]. In short, the protocol included cine imaging in standard short and long axes cine imaging, T2 weighted edema imaging, quantitative T1 and T2 mapping as well as late gadolinium enhancement (LGE) imaging. The latter was performed 10–15 min after application of Gadobutrol 0.01 mmol/kg (Bayer Vital GmbH, Germany). For image analysis, the endomyocardial border in both diastole and systole were traced manually in order to calculate LVEF. The LGE short axis stack was also segmented manually. Scar tissue was identified as a deviation in signal intensity by 5 standard deviations or more from the signal intensity of remote regional tissue, and LV mass, LGE mass and LGE/mass (in %, reflecting infarct size) were calculated. Patients received CMR at baseline after AMI, followed by repeated CMR at six (FU 1) and twelve months (FU 2) to determine an LVEF increase of ≥ 5% as the endpoint of the study (further referred to as short- and long-term functional recovery) [17].

*Statistics.* We used R (version 4.3.2, R Core Team, 2023, Vienna, Austria) with package rms (6.7.0) and Prism (version 10.4.2, GraphPad, San Diego, California). Values are presented as median, mean ± standard deviation or range in parentheses. Student’s *t* test was used to test for differences between LVEF at baseline relative to follow-up. Uni- and multivariate binary logistic regression analyses were applied to determine baseline parameters with prognostic values, with time-point of PET imaging serving as moderating variable. Receiver operating characteristic (ROC) was applied to demonstrate the incremental value of combined parameters. Using QQ plots, box plots, and density plots, outliers were identified for removal. A p-value of less than 0.05 was considered as statistically significant.

## Results

**Fig. 1:**
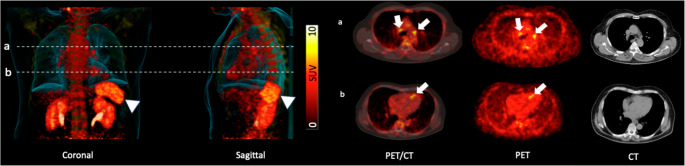
CXCR4-targeted ^68^Ga-PentixaFor PET/CT in a patient after acute myocardial infarction. Dotted lines on the PET/CT maximum intensity projections (MIP, left) in a coronal and sagittal view indicate trans-axial slides on the right. On trans-axial PET/CT and PET in (**a**), an intense PET signal can be observed in mediastinal lymph nodes (arrows), which was not enlarged on the CT portion. In (**b**), the infarct area in the myocardium also exhibits intense in-vivo CXCR4 expression (PET/CT, PET, arrows), while there is no respective finding on CT. On the MIPs (left), radiotracer signal can also be observed in the bone marrow and in the spleen (arrowheads). SUV=standardized uptake value

*Patient’s Characteristics.* Of the 41 patients with single-vessel disease, the affected segments were as follows: 21/41 (51%) with left anterior descending artery, followed by 13/41 (32%) with right coronary artery and the remaining 7/41 (17%) with circumflex coronary artery. Further details on affected vessel segments (proximal, medial, distal) and degree of stenosis can be found in Supplementary Table 2. LVEF at baseline was 50.4 ± 8.7% (range 34–65%), with end-diastolic volume of 167 ± 32 ml, end-systolic volume of 84 ± 25 ml and infarct size of 28 ± 17% (Supplementary Table 3). All patients underwent baseline ^68^Ga-PentixaFor PET/CT. 23/41 patients (56.1%) were allocated to the early (2–4 days) and 18/41 (43.9%) to the late (5–8 days post-AMI) imaging group. Median 5 LN were investigated, with a SUV of 3.61 ± 0.68 and LN ratio of 2.15 ± 0.56. Splenic SUV was 7.23 ± 0.90. Figure 1 and Supplementary Fig. 1 demonstrate PET/CTs of patient post-MI with an intense PET signal in the infarct territory and lymphatic organs, including heart-draining LN, bone marrow and spleen.

*Baseline LVEF and CXCR4 PET-positive LN are associated with functional outcome six months post-MI*,* with only LVEF achieving independent predictive value.* At FU 1, LVEF improved significantly (54.2 ± 7.1%, *P* = 0.0004 vs. baseline; Supplementary Table 3). When investigating the delta LVEF between baseline and FU1, we found a significant correlation with LN uptake (*P* = 0.010; other PET parameters, Ps ≥ 0.109). Follow-up at 6 months (FU 1) was available in 38/41 patients, with 21/38 subjects reaching the endpoint (defined as an LVEF increase ≥ 5%). On univariate analysis, baseline LVEF (Odds ratio (OR) 0.80 [95% CI 0.68–0.90], *P* = 0.002) and LN uptake (OR 0.21 [95% CI 0.10–0.79], *P* = 0.03) reached significance, indicating that lower LVEF and LN uptake were associated with improved outcome. Other PET parameters reflecting lymphatic organ uptake (spleen, OR 1.97 [95% CI 0.91–4.73], *P* = 0.09; bone, OR 2.08 [95% CI 0.82–6.04], *P* = 0.14) and infarct uptake (OR 0.39 [95% CI 0.10–1.38], *P* = 0.15) failed in univariate analysis. We then created a multivariate binary logistic regression model including significant parameters from univariate analysis, along with established clinical parameters of systemic inflammation (WBC, CRP) and infarct size (LGE/mass) as morphological marker of myocardial damage. Baseline LVEF predicted functional recovery independently (OR 0.72 [95% CI 0.54–0.88], *P* = 0.007), while LN uptake failed to reach significance (OR 0.28 [95% CI 0.02–2.07], *P* = 0.24). Time-point of imaging serving as moderating variable also achieved no significance (OR 1.77 [95% CI 0.23–17.54], *P* = 0.59), indicating that timing of PET (late vs. early imaging group) had no impact on functional recovery six months post-AMI. Table 1 provides an overview of uni- and multivariate binary logistic regression at FU 1.

**Table 1:** Uni- and multivariate binary logistic regression analysis six months after acute myocardial infarction (follow-up 1) adjusted for Early/Late Imaging

			Univariate analysis			Multivariate analysis		
Variable			OR	95% CI	P	OR	95% CI	P
Clinical Parameters	CMR	Baseline LVEF	0.80	0.68-0.90	***0.002***	0.72	0.54-0.88	***0.007***
		Baseline LGE	1.03	1.00-1.07	0.09			
		Infarct Size	1.04	0.99-1.09	0.11	0.91	0.81-1.00	0.09
	Laboratory and Clinical Values	NT-proBNP	1.00	1.00-1.00	0.48			
		Peak Troponin T	1.00	1.00-1.00	0.90			
		Peak LDH	1.00	1.00-1.01	0.10			
		Peak CK	1.00	1.00-1.00	0.22			
		CRP	1.24	0.88-1.89	0.25	1.47	0.72-3.62	0.31
		WBC	1.08	0.78-1.51	0.64	1.26	0.68-2.49	0.46
PET		Spleen	1.97	0.91-4.73	0.09			
		Bone	2.08	0.82-6.04	0.14			
		Infarct	0.39	0.10-1.38	0.15			
		LN	0.21	0.10-0.79	***0.03***	0.28	0.02-2.07	0.24
		Early/Late Imaging	0.84	0.23-3.08	0.79	1.77	0.23-17.54	0.59

OR=odds ratio, CI=confidence interval, LVEF=left ventricular ejection fraction, LGE=late gadolinium enhancement, NT-proBNP=N-terminal prohormone of brain natriuretic peptide, LDH=lactate dehydrogenase, CK=creatine kinase, CRP=C-reactive protein, WBC=white blood cell count, LN=lymph node. Infarct size reflected by LGE/mass (%). Early/late imaging refers to the time-point of 68Ga-PentixaFor PET/CT, including an early (2-4 days) or late imaging group (5-8 days) based on tracer availability. Significant values are marked in italic and bold

*Baseline LVEF and CXCR4 PET-derived uptake in the spleen predict functional outcome twelve months after MI.* At FU 2, LVEF of 54.3 ± 7.3% again improved significantly relative to baseline (*P* = 0.006; Supplementary Table 3). When investigating the delta LVEF between baseline and FU2, we found significant correlations with spleen, bone uptake, and LN uptake (*P* ≤ 0.043; infarct, *P* = 0.60). Follow-up at 12 months (FU 2) was available in 36/41 patients, with 21/36 reaching the endpoint (defined as an LVEF increase ≥ 5%). At FU 2, follow-up was available in 36/41 patients, with 21/36 subjects achieving LVEF improvement. On univariate analysis, again baseline LVEF was significant for outcome 12 months post-AMI (OR 0.84 [95% CI 0.73–0.93], *P* = 0.005), along with uptake in the spleen (OR 2.46 [95% CI 1.09–6.36], *P* = 0.04), indicating that lower LVEF and increased splenic PET signal are associated with improved outcome. Other lymphatic organ uptake (bone, OR 2.29 [95% CI 0.86–7.31], *P* = 0.12; LN, OR 0.39 [95% CI 0.09–1.42], *P* = 0.17) and infarct uptake (OR 0.50 [95% CI, 0.13–1.80], *P* = 0.29) failed to reach significance. We then applied the afore-mentioned multivariate binary logistic regression model with significant parameters from univariate analysis and established clinical parameters of inflammation and cardiac damage. Both baseline LVEF (OR 0.73 [95% CI 0.52–0.90], *P* = 0.01) and uptake in the spleen (OR 4.17 [95% CI 1.21–22.35], *P* = 0.04) remained significant for outcome at FU 2, indicating that lower baseline LVEF and increased splenic uptake can identify patients predisposed to long-term functional recovery. Again, timing of PET (late vs. early group) failed to reach significance in this model (OR 4.2 [95% CI 0.52–65.23], *P* = 0.22), supporting the notion that scheduling of molecular imaging within this time frame has no impact on long-term cardiac recovery. Uni- and multivariate binary logistic regressions for FU 2 are presented in Table 2. Supplementary Fig. 2 shows area under the curves for baseline LVEF alone and combined with splenic PET signal.

**Table 2:** Uni- and multivariate binary logistic regression analysis six months after acute myocardial infarction (follow-up 2) adjusted for Early/Late Imaging.

			Univariate analysis			Multivariate analysis		
Variable			OR	95% CI	P	OR	95% CI	P
Clinical Parameters	CMR	Baseline LVEF	0.84	0.73-0.93	***0.005***	0.73	0.52-0.90	***0.01***
		Baseline LGE	1.02	0.99-1.06	0.23			
		Infarct Size	1.02	0.98-1.07	0.30	0.91	0.81-1.00	0.08
	Laboratory and Clinical Values	NT-proBNP	1.00	1.00-1.00	0.46			
		Peak Troponin T	1.00	1.00-1.00	0.29			
		Peak LDH	1.00	1.00-1.00	0.68			
		Peak CK	1.00	1.00-1.00	0.19			
		CRP	1.18	0.83-1.83	0.38	1.68	0.86-3.76	0.15
		WBC	0.93	0.64-1.33	0.68	1.13	0.63-2.11	0.67
PET		Spleen	2.46	1.09-6.36	***0.04***	4.17	1.21-22.35	***0.04***
		Bone	2.29	0.86-7.31	0.12			
		Infarct	0.50	0.13-1.80	0.29			
		LN	0.39	0.09-1.42	0.17			
		Early/Late Imaging	1.04	0.27-3.99	0.95	4.2	0.52-65.23	0.22

OR=odds ratio, CI=confidence interval, LVEF=left ventricular ejection fraction, LGE=late gadolinium enhancement, NT-proBNP=N-terminal prohormone of brain natriuretic peptide, LDH=lactate dehydrogenase, CK=creatine kinase, CRP=C-reactive protein, WBC=white blood cell count, LN=lymph node. Infarct size reflected by LGE/mass (%). Early/late imaging refers to the time-point of ^68^Ga-PentixaFor PET/CT, including an early (2-4 days) or late imaging group (5-8 d) based on tracer availability. Significant values are marked in italic and bold

## Discussion

In timely re-perfused, ST-elevation AMI patients, the CXCR4-directed PET agent ^68^Ga-PentixaFor provided a non-invasive read-out of increased target expression in organs of systemic immune response. Established biomarkers of cardiac damage (infarct size) and systemic inflammation (CRP, WBC) failed to predict myocardial functional recovery. By contrast, cardiac baseline function and CXCR4 uptake in the spleen may be useful to identify patients predisposed to functional recovery 12 months after AMI, indicating that the PET signal provided complementary prognostic value for long-term cardiac outcome. Thus, focusing on distant organs of the systemic immune response, findings of the present trial confirm the usefulness of chemokine receptor PET of extra-cardiac organs post-AMI and tie the concept of whole-body inflammatory imaging to a relevant clinical meaning.

In a previous study, CXCR4 PET signal in the infarct territory was linked to the occurrence of major cardiovascular events [13]. Moreover, a most recent retrospective investigation also focused on the predictive value of ^68^Ga-PentixaFor PET post-MI to determine the predictive value for cardiac functional changes [12]. This previous work investigated a single FU with an observational period of up to 29.5 months after AMI, while the present trial included multiple FU at fixed time-points (6 and 12 months after the acute event), thereby allowing for harmonized outcome comparison within the enrolled study population. Moreover, previous work also exclusively focused on the infarct territory [12]. Results of the present prospective study, however, go beyond the myocardium as the target region, as the CXCR4 PET signal in extra-cardiac lymphatic organs (LN, spleen) was associated with left ventricular recovery after AMI. Thus, we herein demonstrate a potential added clinical value of a whole-body PET approach also imaging distant organs involved in adaptive immunity post-AMI. In this regard, as shown in uni- and multivariate analyses, increased chemokine receptor PET signal in the spleen was linked to long-term functional recovery 12 months after the acute event. Previous work has demonstrated that modulating the CXCR4 / C-X-C motif chemokine ligand 12 axis can deploy splenic regulatory T-cells from the spleen to the infarcted myocardium, which contribute to cardiac repair [18, 19]. With organs expressing CXCR4 in different cell types including lymphocytes, progenitor or natural killer cells [8–10], our molecular imaging approach may therefore provide a non-invasive read-out of the inflammatory cell tracking between the primary site of damage (myocardium) and remote organs of hematopoietic activation (spleen). Nonetheless, PET signal in the spleen exhibited a broad 95% CI, which can also reflect sampling variability inherent to the modest sample size. Relative to the baseline splenic uptake holding predictive potential for long-term outcome twelve months post-MI, we also observed predictive value of LN uptake at least in univariate analysis for short-term cardiac functional changes six months after the acute event. Given the anatomical proximity of heart-draining LN to the primary site of cardiac injury, this phenomenon may be explained by sequential immune activation of first LN, followed by the spleen. LN uptake, however, may also reach significance in future studies including a larger number of subjects as in the present investigation.

There is increasing evidence of substantial benefits of immunomodulating drugs including colchicine or canakinumab for patients after MI [1, 3], while treatment selection was based on systemic biomarkers of inflammation including CRP [3]. While these studies aimed to reduce future vascular events, no specific biomarker is available for identifying patients that might benefit from immunological therapy to improve myocardial function. The observed value of the splenic PET signal for identifying patients predisposed to functional recovery and previous results on CXCR4 expression in the infarct territory for MACE prediction may open avenues for guiding cardiac repair by non-invasive molecular imaging [13]. For instance, recent years have witnessed an increased use of highly specific, targeted drugs such as in-vivo re-programmed chimeric antigen receptor (CAR) T-cells to enhance cardiac repair, which also accumulate in the spleen to provide a target antigen reservoir [20]. Those highly costly targeted interventions necessitate novel approaches for identifying subsets of patients that will most likely benefit from treatment, preferably by providing information on a (sub)cellular level about the absence or presence of the drug target in the myocardium or distant organs, e.g., target antigens in the spleen. Molecular imaging has multiple advantages relative to biopsies that are prone to sampling errors or blood-based systemic biomarkers which cannot provide segregated information on a local cardiac tissue level or on remote lymphatic organs [6]. Relative to morphological imaging, PET can also determine the extent of the underlying pathophysiology, while CMR primarily focus on functional parameters [5]. Nonetheless, in the present study, CMR-derived baseline LVEF and the splenic PET signal were relevant outcome predictors for functional recovery, thereby highlighting the importance of simultaneous assessments of morphological and molecular information for precisely identifying patients with best outcome. Thus, combined devices such as PET/MRI may therefore allow to determine those relevant parameters in a “one-stop-shop” setting, thereby facilitating a broader clinical use of the herein derived findings [21].

Our study has several limitations, including its moderately impaired cardiac function at baseline. However, in the present prospective trial, we still observed substantially decreased LVEF with a lower range of 34%. In this regard, imaging and therapeutic protocols including timely reperfusion therapies have been strictly followed, thereby presenting a cohort reflecting clinical reality along with standardized functional re-assessments at two fixed FU time-points. Such fixed re-examinations, however, do not allow for time-to-event analyses, such as Kaplan-Meier analysis. We acknowledge that the sample size at the 12‑month follow‑up (*n* = 36) is relatively small. However, the attrition rate of only 12.2% is low for a longitudinal clinical study [22] and suggests a homogeneous and stable cohort. This low loss to follow‑up may reduce the risk of attrition bias and may support the robustness of the observed effects. Nonetheless, our preliminary findings should be re-evaluated in a larger multicenter setting. Moreover, due to logistic reasons including synthesis procedures and tracer availability, it was not feasible to conduct CXCR4 PET scans on the same day for every patient, while the respective group sizes were still balanced (early, 56.1% vs. late PET imaging, 43.9%). However, to further address timing of PET, we also adjusted for scan timing without significant results in univariate analyses. Given the fact that chemokine receptors are overexpressed on a broad range of leukocytes, which are fluctuating over time early after AMI [23], it cannot be ruled out that such temporal dynamics may also interfere with severity of the PET signal conducted at different time-points, thereby explaining the missing predictive potential for uptake in the infarct region in our patient population. Nonetheless, unlike in previous investigations [12, 13], the aim of the present prospective study was not to assess the predictive value of the infarct territory, but focused on extra-cardiac organs involved in the immune response imaged with chemokine receptor PET. In this regard, we added to the literature by demonstrating that read-out of LN and splenic uptake within a time window of up to 8 days after AMI can identify patients prone to cardiac functional changes. Future work should also re-assess the predictive value of PET for occurrence of MACE in a prospective setting, thereby providing further clinical value beyond LVEF outcome prediction [13].

## Conclusions

In timely re-perfused, first ST-elevation AMI patients, CMR-derived functional assessment and chemokine receptor PET of LN and spleen identified patients that are predisposed to functional changes during follow-up. Imaging parameters were also more strongly associated with outcome relative to standard markers of cardiac damage or inflammation. As such, findings of the present study tie the concept of whole-body inflammatory imaging to a clinical meaning and may open avenues for image-guided anti-inflammatory therapies based on PET signal strength in lymphatic organs.

## Supplementary Information

Below is the link to the electronic supplementary material.

Supplementary Material 1 (DOCX 1.01 MB)

## Declarations

### Published as preprint

medRxiv 2025.09.04.25334995.
