# Supplementary material for: Molecular imaging of lymphatic organs provides prognostic value after acute myocardial infarction
Source: Eur J Nucl Med Mol Imaging. 2026 Mar 11;53(7):4700–7. doi: 10.1007/s00259-026-07809-2 (PMC13197370; doi:10.1007/s00259-026-07809-2)
Supplement: Supplementary file 1 — Supplementary Material 1 (DOCX 1.01 MB) [file 259_2026_7809_MOESM1_ESM.docx]

**SUPPLEMENTARY TABLES**

| Patient’s Characteristics | |
| --- | --- |
| Weight (kg) | 82±15.4 |
| Height (m) | 1.73±0.10 |
| Age | 59.1±9.4 |
|  | |
| Cardiovascular Risk Factors | |
| Hypertension | 61 % (25 of 41) |
| Type 2 diabetes | 15 % (6 of 41) |
| Obesity | 27 % (11 of 41) |
| Nicotine abuse | 61 % (25 of 41) |
| Hypercholesterinemia | 20 % (8 of 41) |
| Positive family history | 31 % (13 of 41) |
|  | |
| Other Diagnoses | |
| Depression | 12 % (5 of 41) |
| Thyroid dysfunction | 15 % (6 of 41) |
| Pulmonary disease* | 15 % (6 of 41) |
| Silent cerebral stroke | 2 % (1 of 41) |
| Renal dysfunction | 5 % (2 of 41) |
| Prior myocarditis | 2% (1 of 41) |
| Prior COVID-19 infection | 2% (1 of 41) |
|  | |
| Cardiovascular Medication at Discharge | |
| Beta blocker | 90% (37 of 41) |
| ACE inhibitor/AT1 receptor antagonist | 85% (35 of 41) |
| Angiotensin receptor-neprilysin inhibitor | 15 % (6 of 41) |
| Aldosterone antagonist | 15 % (6 of 41) |
| Sodium-glucose transport protein-2 inhibitor | 32% (14 of 41) |
| Statin | 96% (40 of 41) |
| Ezetimibe | 27 % (11 of 41) |
| Acetylsalicylic acid | 100% (41 of 41) |
| Ticagrelor | 32% (13 of 41) |
| Prasugrel | 68 % (28 of 41) |
|  | |
| Laboratory Values | |
| Creatinine (mg/dl) | 1.00±0.18 |
| C-reactive protein (mg/dl) | 2.49±2.83 |
| White blood cell count (x1000/µl) | 8.98±2.53 |
| N-terminal prohormone of brain natriuretic peptide (pg/ml) | 970.68±1194.65 |
| Peak Troponin T (ng/ml) | 3693.74±5756.87 |
| Peak lactate dehydrogenase (U/l) | 605.15±280.17 |
| Peak creatine kinase (U/l) | 1742.78±1430.06 |

**Supplementary Table 1. Patient’s characteristics at baseline.** Laboratory values were obtained at day of PET/CT. *includes chronic obstructive pulmonary disease and asthma.

|  | Culprit lesion | Proximal Segment | | Medial Segment | | Distal Segment | |
| --- | --- | --- | --- | --- | --- | --- | --- |
| Degree of lesion |  | 100% | < 100 % | 100 % | < 100% | 100 % | < 100% |
| LAD | 51 %  (21 of 41) | 8 | 6 | 2 | 5 | 0 | 0 |
| LCX | 17 %  (7 of 41) | 2 | 1 | 2 | 0 | 2 | 0 |
| RCA | 32 %  (13 of 41) | 2 | 1 | 4 | 3 | 2 | 1 |

**Supplementary Table 2. Culprit lesion and degree of stenosis.**

LAD=left anterior descending artery. LCX=circumflex coronary artery. RCA=right coronary artery.

|  | Baseline | FU1 | P-Value^#^ | FU2 | P-Value^#^ |
| --- | --- | --- | --- | --- | --- |
| LVEF (%) | 50.4±8.7 | 54.2±7.1 | 0.0004 | 54.3±7.3 | 0.006 |
| EDV (ml) | 167±32 | 152±33 | 0.036 | 153±35 | 0.108 |
| ESV (ml) | 84±25 | 70±20 | 0.009 | 72±23 | 0.023 |
| LGE/mass (%)* | 28±17 | 18±13 | <0.0001 | 15±12 | < 0.0001 |
| SV (ml) | 83±18 | 82±19 | 0.93 | 81±19 | 0.87 |

**Supplementary Table 3. Cardiac functional parameters derived by cardiac magnetic resonance (CMR) at baseline, follow-up (FU) 1 and FU 2.**

CMR was assessed at baseline after myocardial infarction, followed by repeated CMR at standardized time-points six (FU 1) and twelve months (FU 2). EDV=end-diastolic volume, ESV=end-systolic volume, LGE=late gadolinium enhancement. SV=stroke volume. *Reflects infarct size. ^#^Refers to comparison with baseline.

**SUPPLEMENTARY FIGURES**

**Supplementary Figure 1**

**Whole-body C-X-C Motif Chemokine Receptor 4-targeted PET/CT after myocardial infarction showing uptake in the infarct territory and organs involved in the systemic immune response.** White arrows indicate the infarct territory on trans-axial PET and PET/CT (lower rows) and heart-draining lymph nodes (upper rows). On maximum intensity projection, triangle indicates the spleen.

**Supplementary Figure 2**


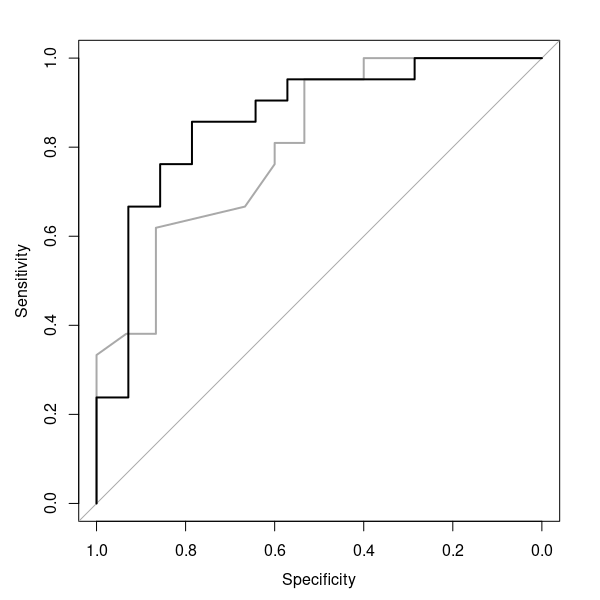


Receiver operating characteristic (ROC) for baseline left ventricular ejection fraction (LVEF) alone (grey) and combination of baseline LVEF and splenic uptake (black), indicating an increased area under the curve for integrating both parameters.
